# Morphologic, Molecular and Clinical Features of Aggressive Variant Prostate Cancer

**DOI:** 10.3390/cells9051073

**Published:** 2020-04-25

**Authors:** Rodolfo Montironi, Alessia Cimadamore, Antonio Lopez-Beltran, Marina Scarpelli, Gaetano Aurilio, Matteo Santoni, Francesco Massari, Liang Cheng

**Affiliations:** 1 Section of Pathological Anatomy, Polytechnic University of the Marche Region, School of Medicine, United Hospitals, 60126 Ancona, Italy; alessiacimadamore@gmail.com (A.C.); m.scarpelli@univpm.it (M.S.); 2 Department of Surgery, Cordoba University Medical School, 14071 Cordoba, Spain; em1lobea@gmail.com; 3 Medical Oncology Division of Urogenital and Head and Neck Tumours, IEO, European Institute of Oncology IRCCS, 20141 Milan, Italy; gaetano.aurilio@ieo.it; 4 Oncology Unit, Macerata Hospital, 62012 Macerata, Italy; mattymo@alice.it; 5 Division of Oncology, S. Orsola-Malpighi Hospital, 40138 Bologna, Italy; francesco.massari@aosp.bo.it; 6 Department of Pathology and Laboratory Medicine, Indiana University School of Medicine, Indianapolis, IN 46202, USA; liang_cheng@yahoo.com

**Keywords:** prostate cancer, aggressive variant, anaplastic prostate cancer, neuroendocrine prostate cancer, aggressive variant prostate cancer, anaplastic prostate cancer

## Abstract

The term aggressive variant prostate cancer (AVPCa) refers to androgen receptor (AR)-independent anaplastic forms of prostate cancer (PCa), clinically characterized by a rapidly progressive disease course. This involves hormone refractoriness and metastasis in visceral sites. Morphologically, AVPCa is made up of solid sheets of cells devoid of pleomorphism, with round and enlarged nuclei with prominent nucleoli and slightly basophilic cytoplasm. The cells do not show the typical architectural features of prostatic adenocarcinoma and mimic the undifferentiated carcinoma of other organs and locations. The final diagnosis is based on the immunohistochemical expression of markers usually seen in the prostate, such as prostate-specific membrane antigen (PSMA). A subset of AVPCa can also express neuroendocrine (NE) markers such as chromogranin A, synaptophysin and CD56. This letter subset represents an intermediate part of the spectrum of NE tumors which ranges from small cell to large cell carcinoma. All such tumors can develop following potent androgen receptor pathway inhibition. This means that castration-resistant prostate cancer (CRPCa) transdifferentiates and becomes a treatment-related NE PCa in a clonally divergent manner. The tumors that do not show NE differentiation might harbor somatic and/or germline alterations in the DNA repair pathway. The identification of these subtypes has direct clinical relevance with regard to the potential benefit of platinum-based chemotherapy, poly (ADP-ribose) polymerase inhibitors and likely further therapies.

## 1. Introduction

Men with CRPCa may evolve an androgen receptor (AR)-independent phenotype, characterized by a rapidly progressive disease course [1,2,3]. This clinically aggressive form is called aggressive variant prostate cancer (AVPCa) [1,2,3]. It is characterized by hormone refractoriness and secondary deposits in different organs [1,2,3]. It often shows a low or absent AR protein expression and is often associated with low serum levels of prostate-specific antigen (PSA) [1,2,3]. In some cases, this aggressive variant expresses markers of neuroendocrine (NE) differentiation [1,2,3]. The transformation to AR-independent AVPCa occurs as a mechanism of adaptive resistance to AR-targeted therapies, including newer AR-targeted treatments [3]. “There is an increase incidence of aggressive variant prostate cancer may be secondary to greater awareness of this entity, patients living longer, and the development of resistance to novel therapies” [3].

It is outside the scope of this contribution to deal with metastatic hormone-sensitive PCa, a tumor that can show clinical, morphological and molecular features of aggressiveness.

## 2. Terminology and Definition of the Aggressive Variant Prostate Cancer

The terminology of the aggressive variant of CRPC lacks consensus among experts.

These aggressive tumors have been called “Anaplastic Prostate Cancer” and “Anaplastic Prostate Carcinoma” (APCa) [1,3,4,5,6]. The term APCa is not accepted by pathologists because the word “anaplastic” is a well-recognized term used to refer to pleomorphic cytology [3]. "Anaplastic" is used to describe clinical features, and does not imply a histologic correlate that might not be present in the morphologic spectrum of this disease [3].

The term “Neuroendocrine prostate cancer” (NEPCa) has also been used to refer to this group of tumors [3]. This term is debated as a way to describe this phenotype with a clinical aggressive course. It implies that a predominantly neuroendocrine histology or small cell carcinoma is present in tissue samples, when it is known that many of such cases do not show typical morphology or immunohistochemical profiles of NE differentiation [3].

The term “Intermediate Atypical Prostate Cancer” (IAPCa) has also been used to refer to CRPCa in men who develop mixed pathologic and molecular features overlapping with NEPCa [5,7]. However, the term is not accepted in the uro-oncology community because it does not reflect the whole clinical, morphological and molecular spectrum of the rapidly progressive disease. 

“Aggressive variant prostate cancer” (AVPCa) [1] is the preferred term used by clinicians to refer to CRPCa with at least one of the following seven features, as recently detailed in a paper by Aparicio et al [8] and Vlachostergios et al [5]:

1. “Histologic evidence of small-cell NEPCa (pure or mixed);
2. The presence of exclusively visceral metastases;
3. Radiographically predominant lytic bone metastases by plain x-ray or CT scan;
4. Bulky (≥5 cm) lymphadenopathy or bulky (≥5 cm) high-grade (Gleason ≥ 8) (i.e., Grade Group ≥ 4) tumor mass in prostate/pelvis;
5. Low PSA (≤10 ng/mL) at initial presentation (prior to ADT or at symptomatic progression in the castrate setting) plus high volume (≥20) bone metastases;
6. Presence of neuroendocrine markers on histology (positive staining of chromogranin A or synaptophysin) or in serum (abnormal high serum levels for chromogranin A or gastrin-releasing peptide (GRP)) at initial diagnosis or at progression, plus any of the following in the absence of other causes:
  - elevated serum LDH (≥2 × ULN),
  - malignant hypercalcemia,
  - elevated serum CEA (≥2 × ULN);
7. Short interval (≤ 6 months) to androgen-independent progression following the initiation of hormonal therapy with or without the presence of neuroendocrine markers” [5,8].

It worth mentioning the updated prognostic model by Halabi et al. [9] for predicting the overall survival in first-line chemotherapy for patients with metastatic CRPCa. This group of authors have identified three risk groups—low, intermediate and high—on the basis of eight prognostic factors, including the Eastern Cooperative Oncology Group performance status, disease site, lactate dehydrogenase (LDH), opioid analgesic use, albumin, hemoglobin, prostate-specific antigen and alkaline phosphatase. This model can be adopted to predict overall survival. Such model could be also used in patients with AVPCa, a clinically aggressive form that represents the evolution of CRPCa. The combination of cabazitaxel/carboplatin in clinically defined AVPCa patients demonstrated a significant 3-month PFS benefit compared to cabazitaxel alone (5.6 months vs. 3.8 months respectively) and a trend towards survival improvement [10].

## 3. Aggressive Variant Prostate Cancer: Morphologic and Immunohistochemical Characterization

**Figure 1:**
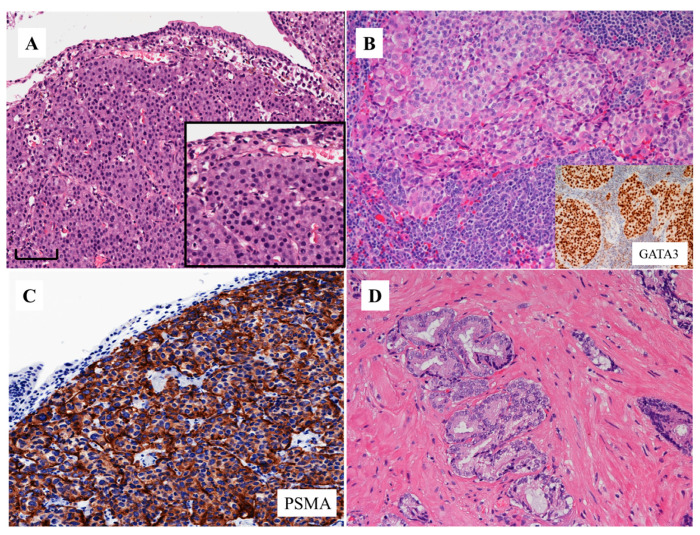
Morphologic appearances of aggressive variant prostate cancer (from a patient identified by feature no. 7 of the list by Aparicio et al [8] and Vlachostergios et al [5]; see text) (**A**) (hematoxylin and eosin stain, H&E) (bar: 100 microns) and urothelial carcinoma of high grade with a GATA3 immunostaining expression (**B**; insert) (H&E stain). The immunohistochemical expression of prostate-specific membrane antigen (PSMA) in the same case of aggressive variant prostate cancer shown in 1a (**C**). Acinar adenocarcinoma of the prostate (**D**) (H&E stain).

**Figure 2:**
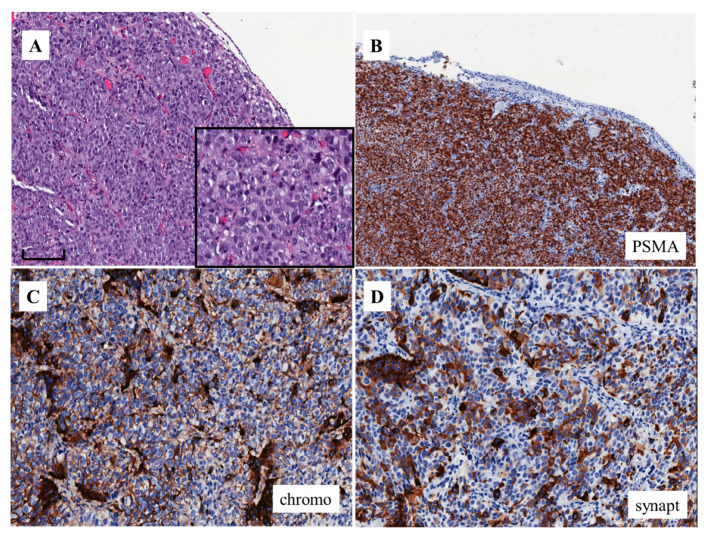
Morphologic appearance of aggressive variant prostate cancer (**A**) (H&E stain) (bar: 100 microns). The immunohistochemical expression of PSMA (**B**) as well as chromogranin A (**C**) and synaptophysin (**D**) in the same case of aggressive variant prostate cancer shown in (**A**) (from a patient identified by feature no. 6 of the list by by Aparicio et al [8] and Vlachostergios et al [5]; see text).

Figure 1 and Figure 2 show two typical cases of AVPCa. Both cases are from the bladder wall infiltrated by a recurrent PCa that has become locally and systemically aggressive. In both cases, it grows under the urothelial lining, which is morphologically normal. The tumor is solid and composed of homogeneous cells with round nuclei, without pleomorphism, with a size at least double that of the urothelium and with prominent nucleoli. The cells form small nests (Figure 1A and Figure 2A).

Based on the morphologic appearance, and without knowing the previous history of the patients, such tumors in the bladder wall could have been considered as high grade invasive urothelial carcinoma because of their similarity to such tumors (Figure 1B). However, both tumors were negative for the immunohistochemical markers usually expressed by urothelial carcinoma, such as GATA3 (Figure 1B, insert) and uroplakins. Both were also negative for PSA and diffusely positive for prostate-specific membrane antigen (PSMA) (Figure 1C and Figure 2B), the latter at the cell membrane and cytoplasmic levels. Such findings favor the diagnosis of prostate cancer [11]. The case represented in Figure 2 was also positive for (i.e., co-expressed) chromogranin A, synaptophysin (Figure 2C,D) and CD56. Such findings favor the diagnosis of PCa with NE differentiation. The other case was negative for these same markers. This means that the first case is an aggressive form, but does not express NE markers.

**Figure 3:**
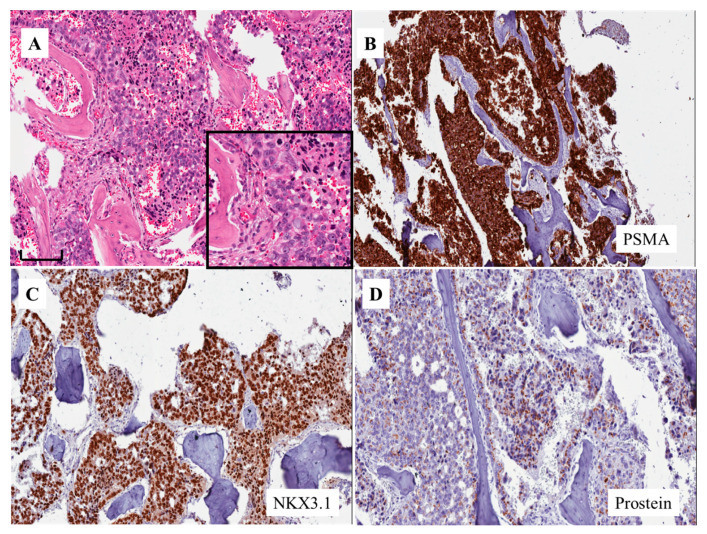
Morphologic appearance of aggressive variant prostate cancer in a bone metastasis (**A**) (H&E stain) (bar: 70 microns). The immunohistochemical expression of PSMA (**B**) as well as NKX3.1 (**C**) and prostein (**D**) in the same case of aggressive variant prostate cancer shown in **A** (from a patient identified by feature no. 3 of the list by Aparicio et al [8] and Vlachostergios et al [5]; see text).

AVPCa is associated with a high frequency of distant metastasis in a typical site (i.e., bone metastasis; being subdivided into lytic or blastic) and/or in atypical sites, such as liver, brain, lung, pleura, mediastinum, peritoneum and adrenal [6]. Figure 3A shows the bone blastic metastasis of a solid tumor morphologically similar to those in the cases of Figure 1A and Figure 2A. The tumor was intensely positive for PSMA (Figure 3B) and co-expressed chromogranin A. The final diagnosis was that of bone metastasis of AVPCa with an NE differentiation.

The three cases presented above, all with a previous history of potent androgen receptor pathway inhibition, exemplify the basic morphology of AVPCa. None of them bears resemblance to typical cases of prostatic adenocarcinoma with acinar or ductal features. There are no attempts to form lumina (Figure 1D). They are more similar to Gleason pattern five. However, in such neoplasms a grading system either based on the Gleason scores or grade groups is not applicable, due to the fact that the tumors have undergone substantial architectural changes to the point that they do not bear any resemblance to typical androgen-sensitive PCa and, as mentioned above, can mimic closely the tumors of other sites, such as urothelial carcinoma.

In such tumors, the final diagnosis depends on the application of immunohistochemical markers that point to the diagnosis of prostate origin, in particular PSMA (see below, i.e., PSMA and aggressive variant prostate cancer), and the exclusion of malignancies from other sites, including the bladder, such as the presence of GATA3 (Figure 1B, insert) and uroplakins. Occasionally, the tumor has undergone such changes that we need to apply additional markers, such as NKX3.1 (Figure 3C) and prostein (Figure 3D), to prove that it is of prostate origin [11]. 

## 4. Questions Related to Aggressive Variant Prostate Cancer

A first question is whether it is possible to distinguish AVPCas with NE differentiation from those without on the basis of the pure morphology, that is, the hematoxylin-eosin-stained sections. The answer is no, because the neoplastic population is quite homogenous in morphology and there is no cellular hint to the presence of NE differentiation, even in cases in which the tumor is composed of cells co-expressing NE and prostate markers. This means that immunohistochemistry has to be performed in all cases of AVPCa to detect an NE differentiation. This is in agreement with the results of the investigation by Labrecque et al [12], showing that molecular profiling identifies five diverse phenotypes based on the expression of well-characterized AR or NE genes: “(i) AR-high tumors, (ii) AR-low tumors, (iii) amphicrine tumors composed of cells co-expressing AR and NE genes, (iv) double-negative tumors, and (v) tumors with small cell or NE gene expression without AR activity” [12].

**Figure 4:**
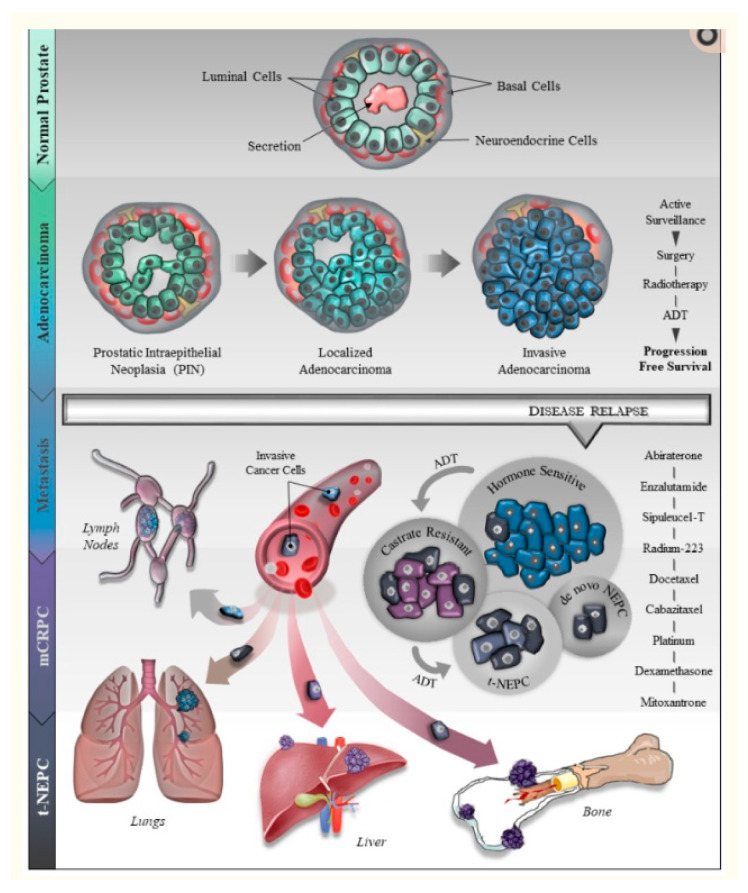
“A generalized overview of prostate cancer (PCa) progression, metastasis, drug resistance and neuroendocrine differentiation (NED). The illustration describes PCa development from normal epithelial cells (Basal, Luminal and NE cells) to prostatic intraepithelial neoplasia (PIN) to localized- and invasive adenocarcinoma. The cartoon depicts several therapeutic regimens used for the treatment of PCa including surgical resection, radiotherapy and androgen deprivation therapy (ADT). After the initial response to ADT, the majority of the patients relapse with resistance to ADT leading to castration resistant prostate cancer (CRPC) with or without metastasis. These patients are further treated with the next-generation ADTs, enzalutamide or abiraterone. During the course of CRPC treatment, about 30% of PCa patients develop a more aggressive and fatal form of the disease called t-NEPC that has very limited therapeutic responses”. Reproduction from [14] Patel GK, Chugh N, and Tripathi M. Cancers (Basel), 2019, under the Creative Commons Attribution License Attribution 4.0 International (CC BY 4.0).

An additional question is whether the morphologic spectrum of NE differentiation in AVPCa can be considered to be much wider. The answer is that the type of tumor described above could represent the intermediate part of a morphologic spectrum of NE tumors, a spectrum that could range from small cells to large cells [1,5,13] (see below: small cell and large cell NE tumors). All such tumors can develop following AR pathway inhibition (Figure 4). This means that “castration-resistant prostate cancer transdifferentiates to treatment-related NE prostate cancer” [2]. In particular, the majority of evidence to date favors “a trans-differentiation model”, i.e., adenocarcinoma cells undergo trans-differentiation into NEPCa cells. There are data showing the role of NE cells in promoting the growth of cancer through endocrine and paracrine factors [3,14].

A further question is whether NE differentiation can be observed in PCas that are still hormone sensitive and therefore not really part of the spectrum of AVPCa. The answer is “yes”, and they have the morphology of mixed acinar and NE adenocarcinoma [13] (see below: focal neuroendocrine differentiation in prostate cancer).

Another question is what kind of molecular features can be detected in the AVPCa without NE differentiation. The answer is that such tumors can be characterized by the presence of specific alterations in the DNA that can have a role from the therapeutic point of view [15] (see below, i.e., mutations to the germline DNA and mutations other than defective DNA repair mechanisms).

In the normal prostate, the epithelial lining of the duct and acini is composed of stem cells, luminal PSA-positive and AR-driven cells and, to a lesser extent, NE cells. A final question is related to what type of cells are mostly represented in AVPCa. The answer is that it has the molecular features of stem cells [16] (see below: basal stem cell signature).

## 5. PSMA and Aggressive Variant Prostate Cancer

PSMA is a type II transmembrane protein with an extracellular C-terminus, a helical transmembrane structure and an N-terminal cytoplasmic tail [17,18]. By immunohistochemistry, PSMA can be seen on the surface of the cell, at the cell membrane level and/or in the cytoplasm. These should correspond to the extracellular domain of the molecule (i.e., the accumulation on the surface of the cell), the transmembrane region (i.e., cell membrane staining) and the cytoplasmic domain (i.e., cytoplasmic staining). The three immunostaining cell patterns should represent “statically” the different stages of the internalization mechanism of the PSMA molecule [19].

Concerning the PSMA aggressiveness of PCa, the PSMA expression increases considerably from focal within the prostate to a more diffuse pattern in metastatic or secondary deposits [17], as seen in the AVPCa cases presented here.

A predominant cell location on the surface of the cell is seen following androgen ablation therapy. An accumulation on the cell surface, enhanced by androgen ablation, could improve cell cancer visualization with imaging techniques [20] and potentially when the PSMA molecules are targeted for imaging and therapy at the same time [17,21].

## 6. Small Cell and Large Cell NE Tumors

NE prostate cancer, including pure small-cell cancer, is very rare at the time of diagnosis (fewer than 1% of cases). It is an increasingly common phenomenon as a treatment-emergent adaptive response under androgen signaling inhibition [12,22,23]. The exact incidence of this entity in the setting of mCRPCa is not known with certainty, with recent studies showing pure small cell carcinoma (SmCC) in 10% to 15% of the specimens [23,24].

### 6.1. Small Cell Carcinoma (SmCC)

**Figure 5:**
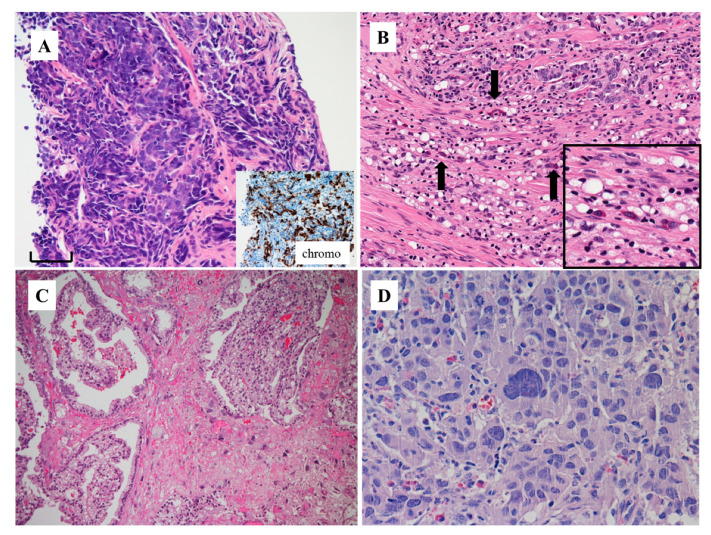
Small cell carcinoma of the prostate (**A**) (H&E stain) (bar: 70 microns). High grade PCa with Paneth cell-like rich areas (arrows) (**B**) (H&E stain). Carcinosarcoma (**C**) (H&E stain9 and pleomorphic carcinoma of the prostate (**D**) (H&E stain).

SmCC represents between 1% and 5% of all prostatic malignancies when mixed adenocarcinoma-neuroendocrine carcinoma are included [13,24]. Many patients have a previous history of a hormonally treated adenocarcinoma. Morphologically, SmCC is identical to small cell carcinoma of the lung [25] (Figure 5A). In approximately 50% of the cases, the tumors are mixed SmCC and adenocarcinoma of the prostate. NE carcinoma should not be assigned a Gleason grade and grade group and must be differentiated from the diffuse growth in Gleason pattern five PCa, based on the fact that the latter shows large cells with a lower N/C ratio and prominent nucleoli.

There are conflicting data as to whether SmCC originating in the prostate is positive for TTF-1, a marker that is usually expressed in SmCC of the lung. A recent study demonstrated that the stain for TTF-1 is positive in approximately 50% of cases in the prostate [13,25]. The same study also showed that P504S/alpha methylacyl CoA racemase and PSMA are better at identifying the prostatic origin of SmCC, although 60% of prostatic SmCCs are negative for all three markers. For small cell carcinoma of unknown primary origin, a positive ERG break-apart fluorescence in situ hybridization test is useful to confirm a prostatic origin [26].

CD44 is a cell-surface molecule that can identify cancer stem/progenitor cells in PCa. Strong and diffuse membrane staining for this marker is seen in 100% of prostatic SmCCs [13] (see below: basal stem cell signature). In conventional PCa, positive staining is seen in rare, scattered tumor cells, whereas CD44 staining is negative in most of the SmCCs originating outside of the prostate [27].

The average survival is less than a year, with 12.5% of patients alive at 5 years [28]. There are studies suggesting that SmCC of the prostate should be treated with the same combination chemotherapy as SmCC in other locations [13].

### 6.2. Large Cell Neuroendocrine Carcinoma (LCNEC)

Few cases of prostatic LCNEC have been described [13,29]. Morphologically, LCNEC is composed of solid sheets and ribbons of cells with abundant amphophilic cytoplasm, large nuclei with coarse chromatin and prominent nucleoli, along with foci of necrosis and brisk mitotic activity. LCNEC may be easily mistaken for Gleason score 5 + 5 = 10 (Grade group 5) PCa and, hence, the likelihood of being underdiagnosed is high [13,29]. LCNEC is positive for chromogranin A, synaptophysin, CD56 and P504S/alpha methylacyl CoA racemase—this last marker is usually expressed in PCa. It is focally positive for PSA and negative AR staining. The prognosis for LCNEC is similar to that of SmCC. Patients with de novo tumors mixed with prostatic adenocarcinoma may respond to ADT and might present a better outcome than those with pure LCNEC or post-ADT LCNEC of the prostate [30]. 

### 6.3. Focal Neuroendocrine Differentiation in PCa

Almost all PCas show focal NE differentiation, in general in the form of only rare or sparse single NE cells, highlighted by NE markers. In 5%–10% of PCas, there are zones with a large number of single or clustered NE cells, either single or clusters [13,24].

Occasionally, NE cells with cytoplasmic eosinophilic granules, resembling the Paneth cells of the gastro-intestinal tract [31] (Figure 5B), are seen in a patchy manner in normal as well as in neoplastic tissues. A recent investigation showed that among cases with Paneth cell-like rich areas resembling high grade PCa, none showed evidence of progression.

The prognostic significance of NE differentiation in primary untreated PCa is unclear, with some studies showing an independent negative effect upon prognosis, whereas others did not confirm such an observation [32].

### 6.4. NE Differentiation and Somatostatin Receptors

An immunohistochemical study investigating the expression of the five subtypes of somatostatin receptors (termed SSTR1 to 5) in PCa has shown that the greatest proportion of cells with strong stainings is seen in SSTR2, mainly in the group of CRPCa with NE differentiation [33]. The cloning of the SSTRs has led to the development of subtype-selective analogues. Among those, the SSTR2-specific somatostatin (SST) analogues octreotide and lanreotide have attracted significant attention. Typing the somatostatin receptor expression in NE tumors is considered to be of great relevance for somatostatin analogue-based diagnostic and therapeutic approaches. The presence of somatostatin receptors on the cancer cell surface may provide a readily available, noninvasive means to identify PCa with NE differentiation with imaging techniques as well as for peptide receptor radionuclide therapy [34].

## 7. Alterations in the DNA

Emerging clinically relevant subcategories in AVPCa include disease that demonstrates not only NE differentiation but also tumors with somatic and/or germline alterations in the DNA repair pathway [15]. “Identification of these subtypes has direct clinical relevance with regard to the potential benefit of platinum-based chemotherapy, poly (ADP-ribose) polymerase inhibitors, and likely further therapies as new therapeutic targets are identified in these groups” [22].

A recent study by Aparicio et al. dealt with AVPCa, defined by them as “a clinically defined subset of the disease that shares virulent and atypical clinical features and chemotherapy sensitivity with the small cell prostate carcinomas” (SmCC) [35]. They observed that clinically defined AVPC shares molecular features with SmCC and is characterized by combined alterations in RB1, Tp53 and/or PTEN. According to the authors, such molecular signature accounts for the “shared clinical features, resistance to AR inhibition and chemotherapy sensitivity and should serve as the foundation for a biologically-defined therapeutically relevant classification of prostate cancer” [35].

Aurora kinase A (AURKA) amplification and overexpression are implicated in the pathogenesis of SmCC [36]. A high frequency of increased nuclear and/or cytoplasmic AURKA expression as well as a 20 q amplification has been observed in 24.1% of AVPCa cases, irrespective of their morphology [35]. The interactome of AURKA is large [37] and includes cell cycle and transcriptional regulators such as AR [38], Tp53 [39], MYC [40] and BRCA1 [41]. For instance, tumor suppressor gene TP53 mutations—responsible for defects in p53, a protein with an antiproliferative role—increases AURKA expression and cooperates with AURKA in the induction of genomic instability and in the regulation of DNA damage repair [42]. Mutations in such genes are considered prognostic biomarkers to help predict aggressiveness. The results from a recent phase II clinical trial evaluating an Aurora kinase inhibitor, Alisertib, in neuroendocrine prostate cancer patients have been published. The six-month radiographic progression-free survival was 13.4% and the median overall survival was 9.5 months, with some exceptional responses [43].

### 7.1. Mutations to the DNA Damage Repair Pathway

Sequencing DNA for somatic mutations requires PCa material—in particular, biopsies—surgical material and circulating tumor cells or circulating tumor DNA in the blood [44]. Somatic mutations may change over time, due to the selective pressure from therapy and genetic instability. Repeat testing of the tumor DNA is needed during the course of the disease. Testing from primary or metastatic sites or blood may help guide treatment options [45]. “Tumor-based testing has the potential to identify germline mutations that have implications for inherited cancer predisposition. If somatic testing identifies a mutation in a gene associated with PCa predisposition (e.g., BRCA), referral to a genetic counselor for confirmatory germline testing is indicated” [15].

Advances in the technology of DNA sequencing have clarified the genomic landscape of PCa, including AVPCa. Tumors possessing DNA repair mutations and mutations of the defective DNA repair pathway are quite common in advanced stages of neoplasia, thus showing a poor prognosis related to these mutations. Between 20%–25% of men with mCRPCa can present mutations in the DNA repair pathway (such as BRCA genes) [46]. The knowledge that PCa harbors mutations in the DNA repair genes has emerged together with the development of PARP inhibitors, i.e., drugs that are designed to target DNA repair-deficient and BRCA-mutated cancer.

A defective DNA repair system can increase the frequency of mutations in the DNA. This is considered a very important element in the development of the antitumor immune response. Mutations in the BRCA2 gene have been observed in melanoma patients with a better response to anti-PD-1 therapy [47]. Such findings support the potential use of the mutation status of DNA repair genes to predict the response to immunotherapy in CRPCa, including AVPCa.

A recent study by Rantapero et al. has investigated the inherited DNA repair gene mutations in men with lethal PCa in Finnish and Swedish populations [48]. The authors have shown that among the lethal cases, a total of 16 potentially damaging protein-truncating variants in DNA repair genes were identified in 15 men (12.3%). Mutations were found in six genes, with CHEK2 (4.1%) and ATM (3.3%) being the most frequently mutated. The same group concluded that “DNA repair genes are strongly associated with lethal aggressive prostate cancer in Sweden and Finland and highlight the importance of population-specific assessment of variants contributing to aggressive prostate cancer aggressiveness” [48].

### 7.2. Mutations other than Defective DNA Repair Mechanisms

Germline mutations in the mismatch repair (MMR) genes (MLH1, PMS2, MSH2, and MSH6) are seen in Lynch syndrome, an inherited condition that predisposes individuals to an increased risk of developing many different types of cancers. Studies have suggested a slight increase in risk for PCa in men with this syndrome [16,49]. HOXB13 G84E is a germline variant associated with an increased risk of developing PCa [50]. It is not associated with an increased disease aggressiveness with certainty. This should influence treatment decision-making.

### 7.3. Liquid Biopsy

Sequencing DNA for somatic mutations can also be done in circulating tumor cells or circulating tumor DNA in the blood [15]. There are emerging techniques that can allow the molecular assessment of tumors in plasma cell-free DNA. This relies on the differences in fragment length in cancer-derived circulating tumor DNA. These techniques are not yet widely used in clinical practice [44].

## 8. Basal Stem Cell Signature

Evidence from several cancers suggests that an increased aggressiveness is associated with the up-regulation of signaling pathways and with the acquisition of properties seen in stem cells [13,16]. Smith et al. developed a gene signature specific for human prostate basal cells that is differentially enriched in various phenotypes of late-stage metastatic PCa [16]. The authors The author purified by Fluorescence Activated Cell Sorting (FACS) purified and transcriptionally profiled basal and luminal epithelial populations from the benign and neoplastic areas of PCa. They showed that the basal cell population is defined by genes associated with stem cell invasiveness and signaling programs. A panel of a 91-gene basal signature, investigated in patients with organ-confined and mCRPCa, showed that metastatic SmCC was molecularly more stem-like than metastatic PCa and organ-confined PCa [15]. In particular, analyses of the normal basal cell and of human small cell gene signatures identified a set of E2F target genes commonly present in prostate SmCC and primary normal basal cells of the prostate [16]. Such information suggests that “aggressive prostate cancer shares a conserved transcriptional program with normal adult prostate basal stem cells” [16].

## 9. Conclusions 

AVPCa refers to AR-independent anaplastic forms of PCa characterized clinically by a rapidly progressive disease course [1]. Biopsies may show the morphologic spectrum and features of ACPCa. The immunohistochemistry of AR signaling markers (i.e., PSA, AR, PSMA, NKX3.1 and prostein) and classic NE markers (i.e., chromogranin, synaptophysin and CD56) may support not only a diagnosis but also the definition of the subtypes, including small cell and large cell NE tumors [11]. An accumulation of PSMA expression on the cell surface, enhanced by androgen ablation, as shown also by immunohistochemistry, could improve cell cancer visualization with imaging techniques and could represent a target for therapeutic purposes [17,21].

Recent investigations have shown that tissue biopsies from patients with AVPCa can provide molecular information that is clinically relevant, including somatic mutations to the DNA damage repair pathway, mutations other than defective DNA repair mechanisms [15] and the basal stem cell signature [16]. “Additionally, liquid biopsies of circulating tumor cells and circulating tumor DNA can inform a patient’s prognosis, predict response to new hormonal therapies, and serve as a discovery platform for precision medicine” [44].

The morphologic spectrum of treatment-related aggressive variants of PCa also includes rare histological variants such as carcinosarcoma (Figure 5C), adenosquamous carcinoma and pleomorphic carcinoma (Figure 5D) [5]. It is outside the scope of this contribution to deal with the morphologic and clinical features of these additional tumors. The interested reader should consult specific publications related to them.

## Abbreviations

AVPCa	Aggressive variant prostate cancer
AR	androgen receptor AR
PCa	prostate cancer
PSMA	prostate-specific membrane antigen
NE	neuroendocrine
CRPCa	castration-resistant prostate cancer
PSA	prostate-specific antigen
APCa	anaplastic prostate cancer or anaplastic prostate carcinoma
NEPCa	neuroendocrine prostate cancer
IAPCa	intermediate atypical prostate cancer”
CT	scan computerized tomography (CT) scan
ADT	androgen deprivation therapy
GRP	gastrin-releasing peptide
LDH	lactate dehydrogenase
CEA	carcinoembryonic antigen
GATA3	GATA binding protein 3
NKX3.1	NK3 homeobox 1
SSTR	somatostatin receptors
BRCA	breast-related cancer antigens
MMR	mismatch repair
HOXB13	homeobox B13
E2F	E2 factor
